# Immunoregulatory Protein Profiles of Necrotizing Enterocolitis *versus* Spontaneous Intestinal Perforation in Preterm Infants

**DOI:** 10.1371/journal.pone.0036977

**Published:** 2012-05-14

**Authors:** Kathy Yuen Yee Chan, Fiona Wan Lun Leung, Hugh Simon Lam, Yuk Him Tam, Ka Fai To, Hon Ming Cheung, Kam Tong Leung, Terence Chuen Wai Poon, Kim Hung Lee, Karen Li, Tai Fai Fok, Pak Cheung Ng

**Affiliations:** 1 Department of Paediatrics, Prince of Wales Hospital, The Chinese University of Hong Kong, Hong Kong; 2 Department of Surgery, Prince of Wales Hospital, The Chinese University of Hong Kong, Hong Kong; 3 Department of Anatomical and Cellular Pathology, Prince of Wales Hospital, The Chinese University of Hong Kong, Hong Kong; University of Florida, United States of America

## Abstract

Necrotizing enterocolitis (NEC) and spontaneous intestinal perforation (SIP) are the most common acute surgical emergencies associated with high morbidity and mortality in preterm infants. We aimed to compare the profiles of immunoregulatory proteins and identify novel mediators in plasma of NEC and SIP infants. We also investigated the expression of target genes in resected intestinal tissues and an enterocyte cell line. Using Cytokine Antibody Array assay, we reported the first comparative profiles of immunoregulatory proteins in plasma of NEC and SIP infants, and showed that dysregulated proteins belonged to functionally diversified categories, including pro- and anti-inflammation, angiogenesis, cell growth, wound healing, anti-apoptosis, cell adhesion and extracellular matrix reorganization. Validation by ELISA confirmed significantly higher concentrations of interleukin (IL)-6, angiopoietin (Ang)-2, soluble type II interleukin-1 receptor (sIL-1RII), and soluble urokinase-type plasminogen activator receptor (suPAR) in NEC infants compared with gestational age-matched control, and a lower level of an epidermal growth factor receptor, secreted form of receptor tyrosine-protein kinase ErbB3 (sErbB3), compared with SIP infants. mRNA expressions of IL1-RII and uPAR were up-regulated in resected bowel tissues from NEC infants, indicating that immunoregulation also occurred at the cellular level. In FHs-74 Int cells, Ang-2, IL1-RII and uPAR mRNA expressions were significantly induced by the combined treatment with lipopolysaccharide (LPS) and platelet activating factor (PAF). Our study provided plasmatic signatures of immunoregulatory proteins in NEC and SIP infants, and demonstrated involvement of multiple functional pathways. The magnitude of changes in these proteins was significantly more extensive in NEC infants, reflecting the different nature of injury and/or severity of inflammation. We speculate that dysregulation of IL-6, Ang-2, IL-1RII and uPAR occurred at both systemic and cellular levels, and probably mediated via LPS and endogeneous PAF signals. Such exaggerated immunologic responses may account for the high morbidity and mortality in NEC compared with SIP patients.

## Introduction

Necrotizing enterocolitis (NEC) and spontaneous intestinal perforation (SIP) are the most frequently encountered surgical emergencies with devastating consequences in preterm infants. Although both conditions may present with intestinal perforation, most neonatologists consider them as two distinct clinical entities with different clinical profile and natural history. Infants with SIP tend to be lower birth weight and have earlier onset of illness compared with NEC infants [1]. A proportion of cases is associated with the use of drugs, such as indomethacin and corticosteroids [2], [3]. At the early stage of presentation, SIP infants have marked clinical stability as well as lacking signs and symptoms suggestive of a severe illness or peritonitis [1]. Radiologic features of pneumatosis intestinalis and portal venous gas are typically absent. Laparotomy reveals isolated intestinal perforation surrounded by normal bowel and usually simple procedure such as direct suturing or resection with primary anastomosis is the treatment of choice. More importantly, histologic investigation commonly shows hemorrhagic necrosis rather than coagulation necrosis [1]. Despite the differences, there are also features common to both conditions. Prematurity is an important and common factor in the development of NEC and SIP. Hypoxia and shock may give rise to regional intestinal hypoperfusion and predispose to mucosal injury resulting in perforation in the terminal ileum, a watershed area of blood supply and the commonest site of intestinal injury in both NEC and SIP patients. In addition, both conditions can be associated with bacterial or fungal invasion into the bloodstream or peritoneal cavity.

**Table 1:** Clinical characteristics of NEC and SIP patients recruited for plasma analyses.

Preterm infants	NEC	NEC Control	*P*-value NEC *vs.* NEC Control	SIP	SIP Control	*P*-value SIP *vs.* SIP Control	*P*-value NEC *vs.* SIP
No of infants, n	13	13		8	8		
Gender, female	6 (46%)	3 (23%)	0.411	1 (12%)	2 (25%)	1.000	0.174
Gestational age, wk	28.6 (27.0–30.0)	28.6 (28.3–28.9)	0.898	25.6 (24.6–32.0)	25.6 (25.5–25.7)	0.957	0.293
Birthweight, g	980 ( 810–1367)	1165 (810–1305)	0.959	793 (631–1414)	813 (726 –875)	0.916	0.311
Apgar score 1 min	6 (4–8)	7 (6–8)	0.348	8 (6–8)	7 (6–8)	0.590	0.212
Apgar score 5 min	8 (7–10)	8 (8–9)	0.895	8 (8–9)	8 (8–9)	0.868	0.766
Age commenced on feeding, days	3 (2–5)	6 (4–10)	0.028*	8 (5–17)	11 (8–13)	0.642	0.188
Age of full feeding, days	34 (13–90)	30 (16–52)	0.738	82 (54–96)	33 (29–46)	0.121	0.195
Postnatal age at onset of illness, days	31 (19–50)	N/A	N/A	9 (7–11)	N/A	N/A	0.002**
Duration of hospitalization, days	138 (27–160)	88 (59–120)	0.778	121 (22–181)	116 (104–134)	0.916	0.717
Died, n	7	0	0.005**	1	0	1.000	0.085
Length of bowel resection, cm	24.5 (22.3–31.8)	N/A	N/A	3.8 (1.8–6.1)	N/A	N/A	0.0003***

Results are expressed as % or median (interquartile range).

Note: N/A = not applicable.

Cascades of inflammatory responses as well as host defense mechanisms against microbials and endotoxin stimulation are likely to be triggered by NEC and SIP. Investigations on immunoregulatory proteins in NEC and/or infection have revealed mediators associated with pro-inflammation [4]–[6], anti-inflammation [5]–[7], and acute proteins [8]. Interleukin (IL)-6, IL-1β, IL-11 and tumor necrosis factor (TNF)-α have been implicated in its pathogenesis and associated with disease severity [4], [6], [9], [10]. To date, there have been no published data on inflammatory mediators in SIP. In addition, profiles of immunoregulatory proteins in NEC and SIP infants have not been systemically evaluated nor compared. The objectives of this study were to compare the profiles of immunoregulatory proteins in plasma of NEC and SIP infants using cytokine array and ELISA analyses. To investigate the association of circulating target proteins with tissue inflammation, damage and repair, we sought to quantify mRNA expressions of these genes in the resected bowel from NEC and SIP patients. To further reveal the involvement of target proteins in enterocytes, we examined their expression levels in human fetal FHs-74 Int cells upon *in vitro* challenge with lipopolysaccharide (LPS) and platelet activating factor (PAF).

## Results

### Clinical characteristics of infants recruited for plasma and tissue protein analysis

The clinical characteristics of NEC and SIP infants recruited for plasma protein analysis are presented in Table 1. Comparing NEC with SIP infants, NEC infants, as expected, had significantly older postnatal age at the onset of disease (*P* = 0.002) and longer length of bowel resection (*P* = 0.0003). There was also a non-significant trend of higher mortality rate in the NEC group (*P = *0.085; Table 1). All control infants had benign gastrointestinal dysmotility [11] and did not require surgery, and all of them survived. Other comparisons between NEC or SIP infants with their respective control groups are summarized in Table 1.

The clinical characteristics of NEC and SIP infants recruited for tissue mRNA analysis are presented in Table 2. As expected, there were significant differences between these subgroups of NEC and SIP infants in postnatal age at the onset of illness (*P* = 0.004), time of disease onset to surgery (*P* = 0.003) and length of bowel resection (*P* = 0.003). Other differences between NEC or SIP infants and surgical control infants are summarized in Table 2.

**Table 2:** Clinical characteristics of NEC, SIP and surgical control patients recruited for mRNA analysis.

Preterm infants	NEC	SIP	Surgical Control	*P*-value NEC *vs.* Surgical Control	*P*-value SIP *vs.* Surgical Control	*P*-value NEC *vs.* SIP
No of infants, n	7	6	6			
Gender, female	3 (43%)	1 (17%)	2 (33%)	1.000	1.000	0.559
Gestational age, wk	28 (26.4–29.3)	25.3 (24.7–30.5)	35.9 (34.0–37.7)	0.032*	0.030*	0.391
Birthweight, g	870 (770–984)	793 (671–1675)	2730 (2073 –2889)	0.015*	0.109	0.668
Apgar score 1 min	6 (3–8)	8 (6–9)	9 (8–9)	0.066	0.276	0.277
Apgar score 5 min	7 (5–9)	8 (8–10)	10 (9–10)	0.060	0.138	0.337
Age commenced on feeding, days	3 (3–14)	8 (8–9)	12 (12–15)	0.514	0.344	0.568
Age of full feeding, days	90 (75–110)	82 (58–95)	92 (29–97)	0.655	0.806	0.724
Postnatal age at onset of illness, days	50 (32–56)	10 (9–12)	4 (1–58)	0.317	0.469	0.004**
Duration of hospitalization, days	153 (104–167)	121 (39–171)	37 (25–84)	0.153	0.575	0.668
Time of disease onset to surgery, h	48 (44–58)	5 (4–8)	24 (24–48)	0.207	0.006**	0.003**
Died, n	3	1	0	0.192	1.000	0.559
Length of bowel resection, cm	25.0 (19.0–30.5)	2.6 (1.4–5.7)	12.3 (6.5–17.6)	0.046*	0.092	0.003**

Results are expressed as % or median (interquartile range).

Note: All surgical specimens were of ileal origin, except 1 SIP specimen was from the descending colon.

### Cytokine array profiles of NEC and SIP patients

**Figure 1:**
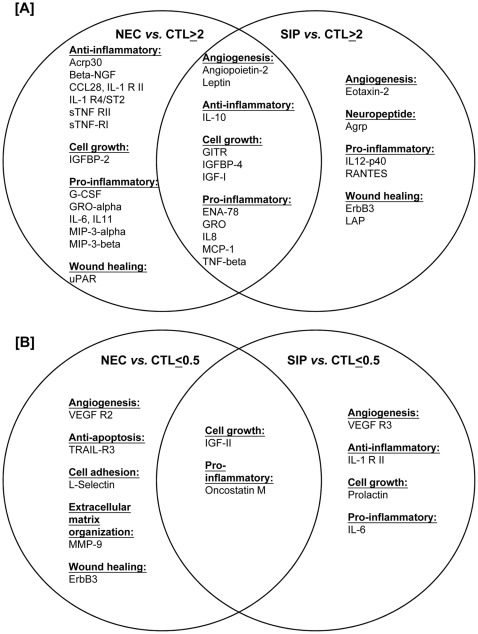
Differentially expressed immunoregulatory proteins in NEC and SIP infants. The plasma profiles of 174 immunoregulatory proteins in infants with NEC (n = 5) and SIP (n = 4) were compared with those in gestational age-matched control infants (n = 5; CTL), using the human Cytokine Antibody Array assay. Using a 2-fold change as the cut- off criterion, differentially up-regulated [Figure 1A] and down-regulated [Figure 1B] proteins in NEC and SIP relative to CTL infants were listed under functional categories.

Using a 2-fold change as the threshold (Table S1), 26 proteins were up-regulated in NEC infants and 17 proteins in SIP infants, compared with controls (Figure 1A). In addition, 7 and 6 proteins were down-regulated in NEC and SIP infants, respectively (Figure 1B). These proteins could be classified into different functional groups of pro-inflammation (14 proteins), anti-inflammation (8 proteins), cell growth (6 proteins), angiogenesis (5 proteins), wound healing (3 proteins), anti-apoptosis (1 protein), cell adhesion (1 protein), extracellular matrix reorganization (1 protein) and neuropeptide (1 protein). Importantly, some dysregulated proteins were common to NEC and SIP, whereas others were more specific to individual diseases (Figure 1). The disparity of clinical manifestations between NEC and SIP could be further reflected in their protein profiles (Figure 1).

### Validation of plasma IL-6, Ang-2, ErbB3, sIL1-RII, and suPAR by ELISA

**Figure 2:**
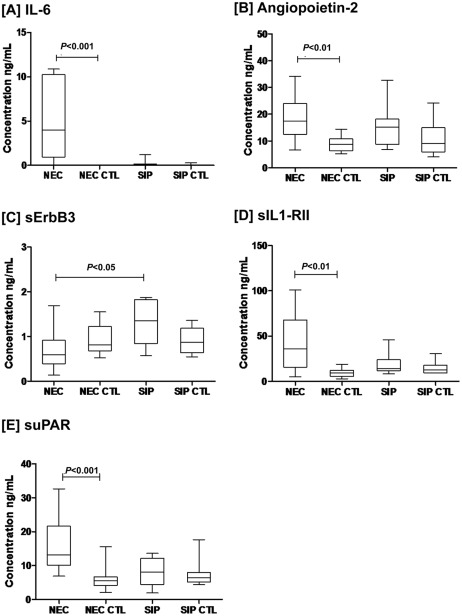
Comparison of plasma levels of target proteins in NEC, SIP and respective control infants by ELISA. Levels of IL-6, Ang-2, sErbB3, sIL1-RII, and suPAR in NEC (n = 13) and SIP (n = 8) infants were quantified by ELISA and compared with those in respective gestational age-matched control (CTL) infants (NEC CTL, n = 13; SIP CTL, n = 8). Levels of IL-6, Ang-2, sIL1-RII and suPAR were significantly higher in NEC infants compared with NEC-CTL (*P*<0.01) (Figure 2A, B, D, E) and SIP infants had significantly higher level of sErbB3 compared with NEC infants (P<0.05) (Figure 2C). Results are presented as median, interquartile range and range.

IL-6, Angiopoietin (Ang)-2, secreted form of receptor tyrosine-protein kinase ErbB3 (sErbB3), soluble type II interleukin-1 receptor (sIL-1RII) and soluble urokinase-type plasminogen activator receptor (suPAR) were targeted for further validation by ELISA (Figure 2). The results were in agreement with those from the cytokine array, showing significant increases of IL-6, Ang-2, sIL1-RII and suPAR in NEC infants compared with NEC-CTL infants (*P*<0.01; Figure 2A, B, D, E). In contrast, levels of these proteins in SIP infants did not significantly differ from SIP-CTL infants. There were also no significant differences between the SIP and NEC group, except that SIP infants had significantly higher plasma level of sErbB3 compared with NEC infants (*P*<0.05; Figure 2C).

In the NEC group, there was a significant positive correlation between Ang-2 and sIL1-RII (*r* = 0.643, *P* = 0.018). Significant inversed correlations were shown between IL-6 and sErbB3 (*r* = −0.56, *P* = 0.046), as well as between suPAR and platelet counts (*r* = −0.791; *P* = 0.001). A non-significant trend between Ang-2 and suPAR was also observed (*r* = 0.511, *P* = 0.074; Figure S1).

### mRNA expression levels of IL-6, Ang-2, ErbB3, IL1-RII, and uPAR in resected intestinal tissues

**Figure 3:**
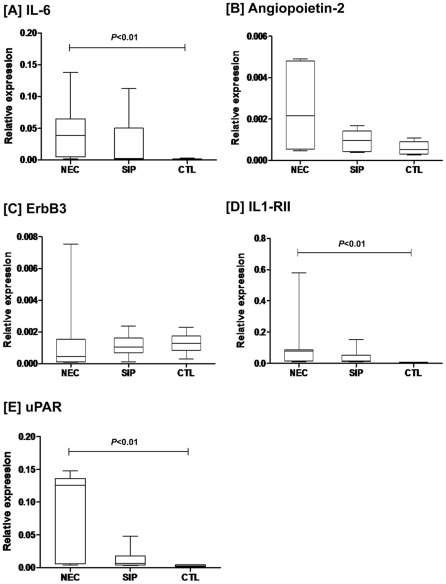
mRNA expression levels of target genes in resected intestinal tissues of NEC and SIP infants. Expression levels of IL-6, Ang-2, ErbB3, IL1-RII, and uPAR in resected intestinal tissues from NEC (n = 7) and SIP (n = 6) infants were quantified by qPCR and compared with surgical control tissues (n = 6). Results showed that IL-6, IL1-RII and uPAR were significantly higher (*P*<0.01) in NEC tissues, compared with surgical control tissues (Figure 3A, D and E). Results are presented as median, interquartile range and range of expression levels relative to β-actin.

To investigate the regulation of target proteins at the tissue level, we quantified mRNA expressions of these genes in resected tissues from NEC (n = 7) and SIP (n = 6) infants (Figure 3). The qPCR results revealed that IL-6, IL1-RII and uPAR expressions were significantly higher (*P*<0.01) in NEC tissues compared with control tissues (n = 6; Figure 3A, D and E). In contrast, mRNA expression levels of these target genes were similar between SIP and control tissues. There were also no significant differences in mRNA expression levels of target genes between NEC and SIP infants.

### mRNA expression levels of IL-6, Ang-2, IL1-RII, and uPAR in FHs-74 Int cell line upon stimulation with LPS and/or PAF

**Figure 4:**
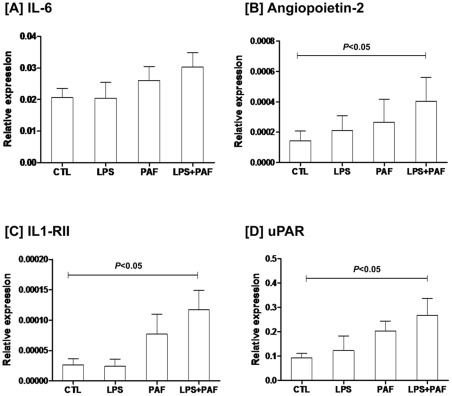
mRNA expression levels of target genes in FHs-74 Int cell line in response to LPS and PAF. FHs-74 Int cells were cultured in the presence of LPS (100 ng/mL) or PAF (25 mM) for 6 h individually or in combination. qPCR analysis of target mRNA expressions showed that combined treatment with LPS and PAF significantly increased levels of Ang-2, IL1-RII and uPAR (n = 4; *P*<0.05), whereas single stimulant, LPS or PAF, did not alter the expression levels. Results are presented as mean and SEM of expression levels relative to β-actin.

Combined treatments with LPS and PAF significantly increased expression levels of Ang-2, IL1-RII and uPAR in FHs-74 Int cells upon culture at a reduced serum concentration (*P*<0.05; Figure 4B, C and D). Treatment with single stimulant, either LPS or PAF, did not significantly alter the expression levels, though a non-significant trend of increase was observed in the PAF cultures.

## Discussion

This study reported the first comparative profiles of immunoregulatory proteins in plasma of NEC and SIP infants and showed that dysregulated proteins belonged to functionally diversified categories. Overall, the number of immunoregulatory proteins and their magnitude of changes appeared more severely altered in NEC infants, especially within categories of pro- and anti-inflammation, compared with SIP infants. In addition, specific anti-apoptosis, cell adhesion and extracellular matrix proteins were only down-regulated in NEC patients, possibly revealing the different nature of bowel injury and/or severity of inflammation compared with SIP. Further validation of selected targets confirmed up-regulation of IL-6, Ang-2, sIL1-RII and suPAR in NEC infants compared with gestational age-matched controls, but the circulating level of sErbB3 was significantly decreased compared with SIP infants. mRNA expressions of IL-6, IL1-RII and uPAR were up-regulated in resected bowel tissues from NEC infants and in accordance with changes in plasma proteins. This indicated that immunomodulation had occurred at the tissue and cellular levels of the affected bowel, which could have contributed to their corresponding increased levels in the plasma. In a model of human fetal enterocytic cell line FHs-74 Int, Ang-2, IL1-RII and uPAR expressions were significantly induced by the combined treatment with LPS and PAF, again suggested that these genes could act synergistically and play a pivotal role in disease development and progression of NEC.

Using cytokine array as a hypothesis-free approach, we provided specific profiles of plasma immunoregulatory proteins in NEC and SIP infants. Our findings are in line with other studies on cytokines, showing regulation of pro- and anti-inflammatory, and repair-associated proteins in NEC infants, including IL-6, IL-8, IL-10, TNF, ENA-78, matrix metalloprotease-9, GRO, MCP-1 and IGF-1 [4]–[7], [12]–[15]. We have also discovered novel immunoregulatory proteins such as Ang-2, ErbB3 and uPAR, which have not been previously described in NEC or SIP. Importantly, there have not been any comprehensive data on immunoregulatory proteins reported in SIP. Our plasmatic protein profile thus represents the basic information platform for further mechanistic investigation of this disease.

Despite sharing many common dysregulated immunomodulatory proteins in the two conditions, some appeared to be more specific to either NEC or SIP, thus reflecting differences regarding the etiology, pathophysiology and severity of inflammation of the two conditions. We validated IL-6, Ang-2, IL1-RII and uPAR in their soluble forms as well as in intestinal tissues and in a fetal enterocyte model, suggesting that the systemic dysregulation of these proteins not only occurred at the plasma level, but also at the cellular level of inflamed bowel tissues and enterocytes. The evidence suggested up-regulation of IL-6, Ang-2, sIL1-RII and suPAR in NEC infants, whereas sErbB3 was significantly higher in the plasma of SIP compared with NEC infants. Despite changes of immunoregulatory proteins were observed in the plasma of SIP patients, the targeted gene levels in the bowel specimens were not different from tissues of surgical control subjects. This might reflect minimal intestinal inflammation due to the localized nature of bowel perforation. The exaggerated up-regulation of immunomodulatory proteins in NEC infants represented imbalance of the inflammatory cascade, which could lead to necrosis, apoptosis and severe tissue damage. This could account for the high morbidity and mortality rate in NEC compared with SIP infants. Of clinical interest, further investigation on validating these dysregulated proteins in a cohort study, either individually or in combination as diagnostic biomarkers, might reveal their usefulness for early differentiation of NEC from SIP patients.

Increased production of the pleiotropic cytokine IL-6 is the hallmark of inflammatory diseases and sepsis. The magnitude of increase reflected the severity of inflammation and has been associated with adverse outcomes such as disseminated intravascular coagulation, multiple organ failure and infant death [4], [9], [14], [16], [17]. Thus, the marked up-regulation of IL-6 and other pro-inflammatory mediators (Figure 1) could provide an explanation for the substantially higher mortality in NEC compared with SIP infants. It has also been suggested that the imbalance of pro-inflammatory to anti-inflammatory cytokines such as a persistently high IL6/IL-10 ratio could predict increased mortality of critically ill adult and neonatal patients [16], [18]. We analyzed this ratio in both NEC and SIP infants and demonstrated that a non-significant trend (*P* = 0.158; Figure S2) existed between those who survived (n = 13) and died (n = 8). It is envisaged that a larger sample size with longitudinal monitoring would be required to fully address the prognostic value of IL-6/IL-10 ratio in surgical infants. IL-6 has been reported to regulate the severity of LPS-driven pro-inflammatory responses via STAT3 and cross-talk between JAK/STAT and toll-like receptor pathways [19]. PAF induced expression of IL-6 was also observed in different cellular systems including adult leukocytes [20], [21], endothelial cells [22] and gut mucosa in a rat model of intestinal damage [23]. Upregulation of IL-6 mRNA was detected in resected tissues from NEC patients, but not in FHs-74 Int enterocytes upon *in vitro* stimulation by LPS and PAF. This observation suggests possible presence of multiple cellular sources of IL-6 production such as endothelial cells and infiltrated leukocytes in inflammed bowel tissues.

Ang-2, an endothelium-specific growth factor, is known to be upregulated in sepsis and inflammatory bowel diseases (IBD) such as Crohn's disease and ulcerative colitis in adults [24], [25]. Ang-2 is involved in angiogenesis and plays a key role in the pathogenesis of IBD [25]. It is positively associated with pro-inflammatory mediators IL-6, IL-8 and TNF-α in septic patients [26]. Ang-2 disrupts vascular quiescence by antagonizing the protective Tie-2 signaling [27], resulting in vascular leakage [24]. Ang-2 also activates neutrophils and enhances PAF synthesis in human endothelial cells [28]. We speculate that Ang-2 may further aggravate the inflammatory cascade and contribute to the pathophysiology of NEC by destabilizing vascular endothelium and increasing vascular leakage.

ErbB3 is expressed in epithelial cells throughout the gastrointestinal tract [29] and sErbB3 is the secreted form of ErbB3 receptor. ErbB3 binds to the ligand, heregulin and blocks it from binding to the cell surface receptor for induction of cell proliferation and differentiation [30]. To date, most studies have associated ErbB3 and sErbB3 with breast and prostate cancers [31], [32] and there has been no report coupling these mediators with systemic inflammatory or gastrointestinal diseases in human. In SIP infants, sErbB3 might inhibit ErbB3 receptor activation on intestinal epithelial cells, thereby limiting cell proliferation and differentiation. This could potentially increase the risk of intestinal perforation.

sIL-1RII is an IL-1β scavenger which negatively regulates the pro-inflammatory signals of IL-1. sIL-1RII is known to be elevated in plasma of septic patients, especially the critically-ill [33], [34]. LPS, TNF-α and other chemoattractants could mediate the release of sIL-1RII [35], [36]. It has been suggested that local shedding of sIL-1RII may decrease colonic inflammation in Crohn's disease [37]. Similarly, it would be plausible that sIL-1RII was released from the site of NEC as a mechanism to regulate and dampen acute reactions of the inflammatory cascade.

Leukocyte is the major site of suPAR production and it functions as a scavenger for inhibiting uPAR signaling, coordinating extracellular matrix proteolysis, promoting cell migration, adhesion and survival [38], [39]. An increase in circulating suPAR level has been implicated in inflammation and sepsis [40], and it has been suggested as a prognostic biomarker of disease severity and fatality in patients with bacteremia [41], [42]. These observations are in accordance with our results that uPAR was markedly increased in the plasma of NEC but not in SIP infants. The significantly higher expression of uPAR in resected intestinal tissues of NEC compared with those of SIP would be consistent with its pivotal role in leukocyte recruitment and adhesion, as well as matrix remodeling.

PAF and LPS are frequently used as stimulants in experimental NEC models as they are key mediators in the pathogenesis of NEC in premature infants [43], [44]. In human enterocytes, LPS is recognized by toll-like receptor (TLR)-4 and involves in activating transcription of pro-inflammatory cytokines IL-8 and TNF [45]. In the current mRNA analysis, we observed that three target genes were significantly upregulated upon combined, but not with individual LPS or PAF treatment, indicating that these two factors could act synergistically in regulating tissue and circulating immunoregulatory proteins. Our findings pointed to multiple levels of gene dysregulations involving proinflammation, anti-inflammation, cell repair and angiogenesis, which might contribute to the pathophysiology of NEC. We propose that LPS and PAF could activate the inflammatory cascade, leading to exaggerated production of pro-inflammatory proteins such as TNF-α and MMP, which in turn stimulate expressions of other mediators, including Ang-2, suPAR and sIL-1RII [26], [35], [36]. The overall mechanism, however, could be highly complex as these proteins are inter-regulatory and may exert positive and negative feedbacks at different levels of the inflammatory cascade. However, it is important to acknowledge that the FHs-74 Int cell model represents only a single cell type amongst multiple cell moieties in the *in vivo* intestinal system, which also harbors the microbiome and infiltrating leukocytes capable of interfering with regulatory pathways.

In summary, our study provided the plasmatic signatures of immunoregulatory proteins in NEC and SIP infants, and demonstrated that multiple mechanistic pathways have been involved. The exaggerated response profile in NEC infants represented an intense activation of the inflammatory cascade and could account for the cytokine imbalance resulting in the high mortality rate observed in NEC compared with SIP infants. Further quantitative analysis of IL-6, Ang-2, sIL-1RII and suPAR revealed regulations at both systemic and cellular levels, possibly mediated by bacterial endotoxin LPS, endogenous PAF and early pro-inflammatory cytokines. Dysregulated angiogenesis and cell repair pathways could eventually contribute to tissue damage and cell death. Our results, thus, provide insights for future investigations. Longitudinal monitoring of novel plasma immunoregulatory proteins and their kinetics could reveal their associations with disease progression. This would allow evaluation of their suitability as diagnostic biomarkers for early differentiation of NEC and SIP, as well as for explaining and predicting the severity and prognosis of these conditions.

## Materials and Methods

### Experimental design and patient population

A total of 13 NEC and 8 SIP infants requiring surgical intervention were recruited into the study within a 50-month period starting in January 2007. All cases of NEC with histologic confirmation were classified by the modified Bell's criteria to have Stage 3 disease. Plasma and tissue samples from these infants were investigated and compared with an equivalent number of control infants.

In the first phase of the study *(i.e.*, within the first 18 months of patient recruitment), we screened and compared the plasma profile of 174 immunoregulatory proteins in infants with NEC (n = 5), SIP (n = 4) and gestational age-matched controls (CTL; n = 5) using the human Cytokine Antibody Array. CTL infants had clinical features suggestive of gastrointestinal dysfunction but were subsequently diagnosed to have benign gastrointestinal dysmotility of prematurity [11] without NEC, SIP or septicemia. Based on Cytokine Array data, potential target proteins were selected on criteria of their relative expression levels, novelty and reversed trend of changes in NEC *versus* SIP. Subsequently, levels of these target proteins were validated by ELISA in all 13 NEC and 8 SIP infants by comparing with their respective CTL infants (NEC-CTL, n = 13; SIP-CTL, n = 8).

In the second phase of the study, we investigated the regulation of target proteins at the tissue level in the same cohort of NEC and SIP infants. Tissue specimens were unavailable for mRNA analysis in: (i) fatal “open and close” cases of NEC (n = 3); (ii) full thickness necrotic specimen (n = 1); (iii) cases with no tissue collection (n = 2); and (iv) SIP infants with primary suturing of perforation (n = 2). mRNA expressions in affected intestinal tissues from NEC (n = 7), SIP (n = 6) and an independent group of surgical control infants (n = 6) were compared by quantitative polymerase chain reaction (qPCR). The surgical control infants were not affected by NEC, SIP nor septicemia, but underwent abdominal surgery due to congenital small bowel atresia (n = 4) and anatomical obstruction (n = 2). These infants were of older gestational age (medium 35.9 weeks, range 34.0–37.7 weeks). All tissue specimens from NEC, SIP and surgical control infants were of ileal origin, except that one specimen of the SIP group was obtained from the descending colon.

In the third phase, the response of selected genes to bacterial endotoxin LPS and stimulant PAF at the mRNA expression levels was determined in the model of human normal fetal enterocyte cell line FHs-74 (n = 4).

Blood samples (0.5 mL) were collected 1–6 h immediately before surgery. We standardized blood sampling to be performed immediately after the decision of surgery was confirmed, as it represented the time when infants were most sick and surgery was imminent and unavoidable. All blood samplings coincided with collections for clinical testing so as to minimize disturbance to the infants. Clinical characteristics of all NEC and SIP infants, as well as those provided surgical specimens are described in Table 1 and 2, respectively. One of 8 SIP infants received dexamethasone on the day of surgery, whereas no NEC patients were treated with systemic corticosteroids or non-steroid anti-inflammatory drugs.

### Protein analysis by cytokine antibody array and enzyme-lined immunosorbent assays (ELISA)

The plasma fraction was separated by centrifugation (1900 *g* for 10 min) at 4°C and stored in 50 µL aliquots at −80°C until analysis. Relative concentrations of immunoregulatory proteins were measured by the Human Cytokine Antibody Array C Series 2000 kit according to the manufacturer's protocol (RayBiotech, Norcross, GA, USA). The 3 membrane arrays 6, 7 and 8 (catalogue number: AAH-CYT-6, AAH-CYT-7, AAH-CYT-8 respectively) covered 174 immunoregulatory proteins. The signal intensity was detected by enhanced chemiluminescence (ECL), (Amersham Biosciences, Little Chalfont, UK) and exposure to X-ray film (Kodak, Rochester, NJ). Quantification of spots was then performed using the GS-700 Imaging Densitometer and the QuantityOne software (BioRad, Richmond, CA, USA). The mean normalized densitometric value from duplicates of each immunoregulatory molecule was obtained using the RayBio® Analysis Tool with background subtraction. Results are expressed as normalized intensities.

Selected protein targets, IL-6, Ang-2, sErbB3, sIL-1RII and suPAR, were quantified in plasma samples of all recruited NEC (n = 13) and SIP (n = 8) infants, and respective CTL (NEC-CTL, n = 13; SIP-CTL, n = 8) by ELISA kits (R&D Systems, Minneapolis, MN, USA and Raybiotech) according to the manufacturer's instruction.

### Measurement of mRNA expression levels in intestinal tissue by qPCR

Immediately after resection, tissue specimens were rinsed with cold phosphate-buffered saline, snap-frozen in liquid nitrogen and then stored at −80°C until tissue homogenization. Total RNA was extracted using TRIZOL reagent (LifeTechnologies, Gaithersburg, MD) and RNeasy mini kit (QIAGEN, GmbH, Hilden, Germany). Gene expression levels of IL-6, Ang-2, ErbB3, IL-1RII and uPAR were quantified and compared with those in surgical control tissues by qPCR using pre-designed or custom-designed TaqMan assays (Applied Biosystems, Foster City, CA). Amplification was performed for 40 cycles with denaturation at 95°C for 15 sec, annealing at 60°C for 1 min. The emission intensity was detected by the ABI 7300 Real-Time PCR System (Applied Biosystems). The average threshold cycles (Ct) were used to calculate the expression ratios relative to β-actin (Applied Biosystems).

### *In vitro* stimulation of human cell line FHs-74 Int by LPS and PAF

The normal fetal intestinal FHs-74 Int cell line (American Type Culture Collection, ATCC, Rockville, MD, USA) was maintained in Hybri-Care Medium 46-X (ATCC) supplemented with 30 ng/mL human epidermal growth factor (R&D Systems) and heat-inactivated fetal bovine serum (FBS, 10%), penicillin G (100 IU/mL) and streptomycin (100 µg/mL) (Invitrogen, Carlsbad, California) at 37°C in a humidified incubator containing 5% CO_2_. FHs-74 Int cells (1.5×10^5^/mL) were cultured for 24 h with reduced FBS at 5% and then treated with LPS (100 ng/mL; Sigma, Saint Louis, MO, USA) or PAF (25 mM; Biomol Research Laboratories, Plymouth Meeting, PA, USA) for 6 h individually and also with these two stimulants in combination. mRNA expression levels of target genes IL-6, Ang-2, IL-1RII and uPAR were determined by qPCR.

### Statistical analysis

This was performed using the GraphPad PRISM program version 5.02 for Windows (Graphpad Software, San Diego, CA, USA) and SPSS (Version 17, Chicago, IL). Clinical data of NEC, SIP and respective control groups were evaluated by the Fisher's exact test and Mann Whitney U test as appropriate. Expression levels of target proteins between NEC or SIP and their respective control groups, as well as between NEC and SIP in plasma and resected intestinal tissues were compared by the Kruskal-Wallis test. Data on mRNA expression of target genes in the intestinal cell line upon treatments with LPS and PAF were analysed using the Friedman test, followed by the post-hoc Dunn's comparison. Linear correlations between plasma proteins, CRP and platelet counts were calculated using the Spearman's correlation test. Results are expressed as median and interquartile range, or as mean and standard error of the mean (SEM). A *P* value of ≤0.05 is considered statistically significant.

### Ethics statement

All blood and tissue samples were collected with written parental consent and in accordance with procedures approved by the Ethics Committee for Clinical Research, The Chinese University of Hong Kong.

## Supporting Information

Figure S1 **Correlation between target proteins in NEC infants.** Plasma levels of IL-6, Ang-2, sIL1-RII, suPAR and sErbB3 in NEC (n = 13) infants were analyzed by the Spearman's correlation test, which showed a significant positive correlation between [A] Ang-2 and sIL1-RII, a significant inverse correlation between [B] IL-6 and sErbB3, as well as [C] suPAR and platelet count. A non-significant trend existed between [D] Ang-2 and uPAR.
(TIF)

Figure S2 **IL-6/IL-10 ratio in NEC and SIP patient subgroups.** The ratio of IL-6/IL-10 protein levels was analyzed on all plasma samples from NEC and SIP patients (n = 21). There was a non-significant trend (*P = *0.158) of higher IL-6/IL-10 ratio on combined NEC and SIP patients who died (n = 8; median IL-6/IL-10 ratio: 3.52; interquartile range: 2.83–7.20), compared with those who survived (n = 13; median IL-6/IL-10 ratio: 1.63; interquartile range: 0.57–5.29).
(TIF)

Table S1 **Comparison of relative protein levels in plasma of NEC, SIP and control infants.**
(DOC)
