# Supplementary figures and images for: Immunoregulatory Protein Profiles of Necrotizing Enterocolitis versus Spontaneous Intestinal Perforation in Preterm Infants
Source: PLoS One. 2012 May 14;7(5):e36977. doi: 10.1371/journal.pone.0036977 (PMC3351425; doi:10.1371/journal.pone.0036977)

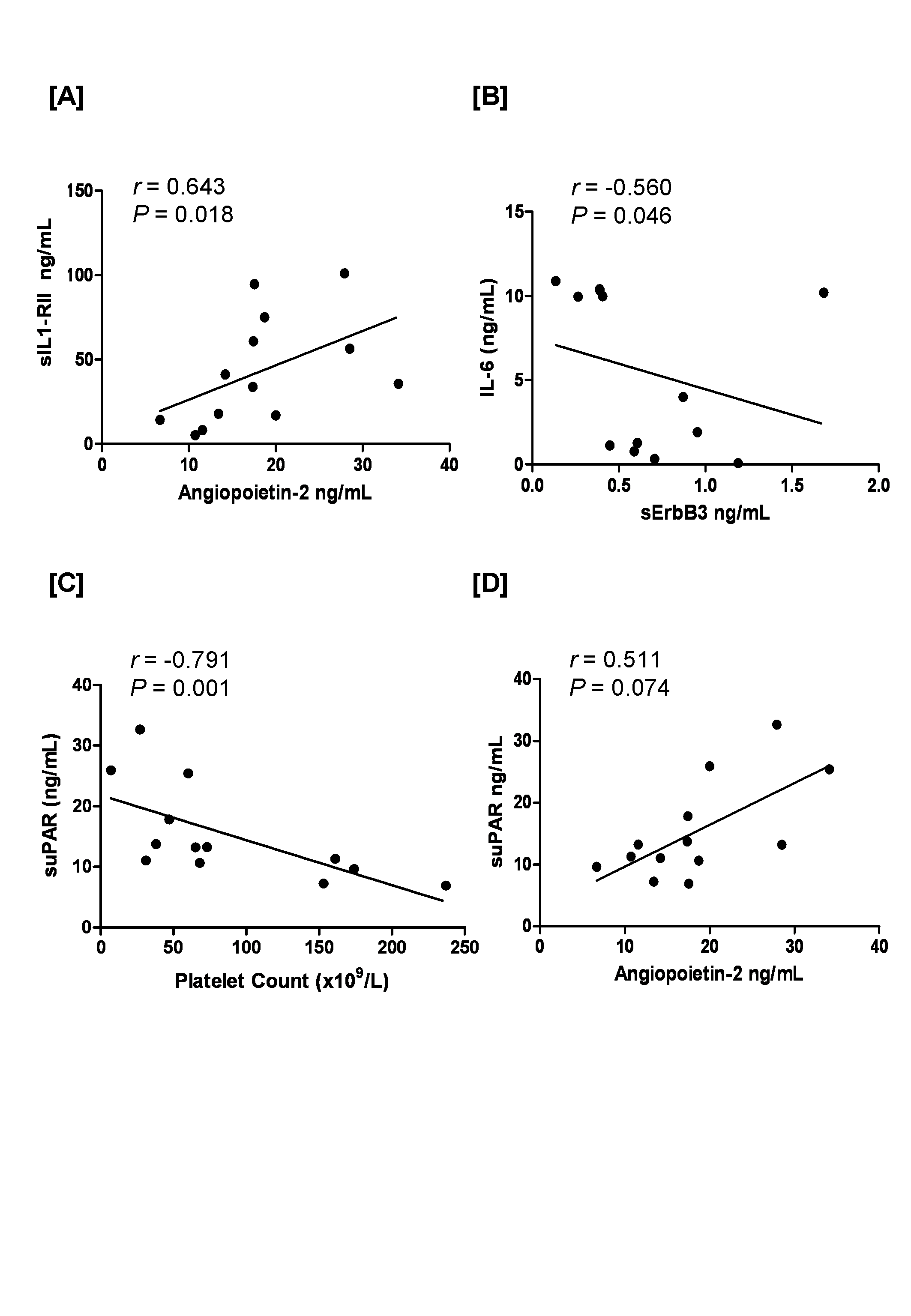

Supplement: Figure S1 — Correlation between target proteins in NEC infants. Plasma levels of IL-6, Ang-2, sIL1-RII, suPAR and sErbB3 in NEC (n = 13) infants were analyzed by the Spearman's correlation test, which showed a significant positive correlation between [A] Ang-2 and sIL1-RII, a significant inverse correlation between [B] IL-6 and sErbB3, as well as [C] suPAR and platelet count. A non-significant trend existed between [D] Ang-2 and uPAR. (TIF) [file pone.0036977.s001.tif]

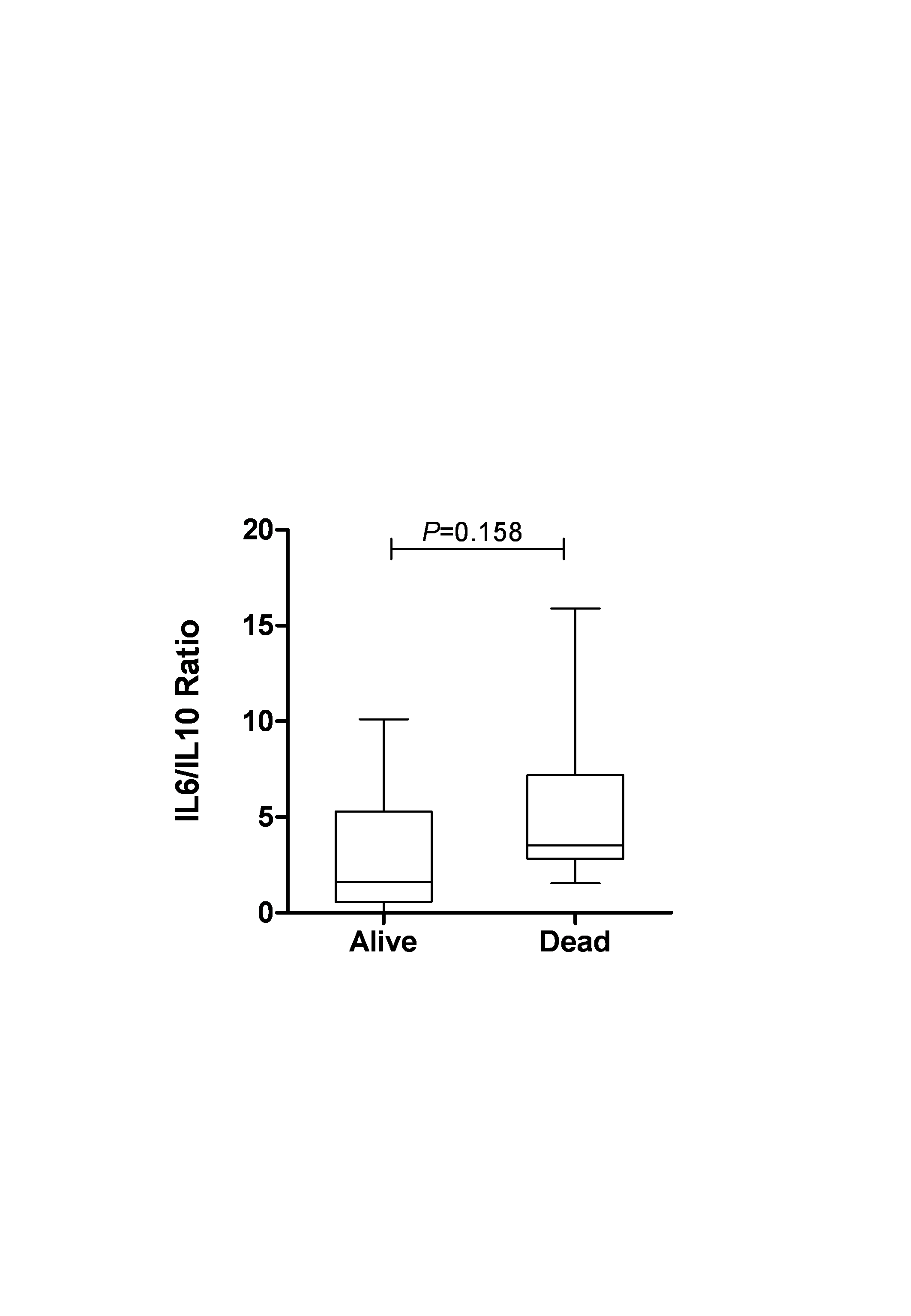

Supplement: Figure S2 — IL-6/IL-10 ratio in NEC and SIP patient subgroups. The ratio of IL-6/IL-10 protein levels was analyzed on all plasma samples from NEC and SIP patients (n = 21). There was a non-significant trend (P = 0.158) of higher IL-6/IL-10 ratio on combined NEC and SIP patients who died (n = 8; median IL-6/IL-10 ratio: 3.52; interquartile range: 2.83–7.20), compared with those who survived (n = 13; median IL-6/IL-10 ratio: 1.63; interquartile range: 0.57–5.29). (TIF) [file pone.0036977.s002.tif]
