# Supplementary material for: Immunoregulatory Protein Profiles of Necrotizing Enterocolitis versus Spontaneous Intestinal Perforation in Preterm Infants
Source: PLoS One. 2012 May 14;7(5):e36977. doi: 10.1371/journal.pone.0036977 (PMC3351425; doi:10.1371/journal.pone.0036977)
Supplement: Table S1 — Comparison of relative protein levels in plasma of NEC, SIP and control infants. (DOC) [file pone.0036977.s003.doc]

**Table S1: Comparison of relative protein levels in plasma of NEC, SIP and control infants.**

| **Immunoregulatory proteins** | **Fold change** | | |
| --- | --- | --- | --- |
|  | **NEC *vs.* CTL** | **SIP *vs.* CTL** | **NEC *vs.* SIP** |
| IL6 | 80.80 | 0.18 | 458.74 |
| MCP-1 | 29.23 | 7.97 | 3.67 |
| IL10 | 25.62 | 25.46 | 1.01 |
| GRO | 17.03 | 4.71 | 3.62 |
| IL8 | 13.41 | 5.92 | 2.26 |
| MIP-3-alpha | 10.57 | 1.18 | 9.00 |
| IGFBP-4 | 5.62 | 4.72 | 1.19 |
| Leptin | 5.51 | 6.51 | 0.85 |
| Angiopoietin-2 | 4.81 | 2.99 | 1.61 |
| IL1-RII | 4.49 | 0.15 | 30.16 |
| IGF-I | 3.69 | 48.91 | 0.08 |
| sTNFRII | 3.28 | 1.96 | 1.68 |
| sTNFRI | 2.99 | 1.51 | 1.98 |
| GITR | 2.45 | 2.13 | 1.15 |
| TNF-beta | 2.44 | 4.26 | 0.57 |
| ENA-78 | 2.36 | 2.13 | 1.11 |
| GRO-alpha | 2.31 | 1.00 | 2.31 |
| uPAR | 2.28 | 1.15 | 1.98 |
| Acrp30 | 2.22 | 1.63 | 1.37 |
| IGFBP-2 | 2.20 | 0.83 | 2.64 |
| CCL28 | 2.17 | 1.43 | 1.51 |
| Beta-NGF | 2.13 | 1.28 | 1.66 |
| IL1R4/ST2 | 2.11 | 1.57 | 1.34 |
| GCSF | 2.10 | 1.93 | 1.09 |
| IL11 | 2.09 | 1.40 | 1.49 |
| MIP-3-beta | 2.03 | 1.21 | 1.68 |
| AgRP | 1.96 | 2.17 | 0.90 |
| IL12-p40 | 1.90 | 2.00 | 0.95 |
| VEGFR3 | 1.17 | 0.44 | 2.66 |
| Eotaxin-2 | 1.01 | 4.57 | 0.22 |
| LAP | 0.75 | 2.07 | 0.36 |
| Prolactin | 0.63 | 0.32 | 2.00 |
| RANTES | 0.58 | 2.76 | 0.21 |
| L-Selectin | 0.45 | 1.19 | 0.38 |
| ErbB3 | 0.43 | 2.55 | 0.17 |
| VEGFR2 | 0.42 | 0.54 | 0.78 |
| OncostatinM | 0.33 | 0.30 | 1.09 |
| TRAILR3 | 0.28 | 0.55 | 0.52 |
| MMP9 | 0.09 | 0.79 | 0.12 |
| IGFII | 0.05 | 0.03 | 1.32 |
